# A case of postpartum spontaneous coronary artery dissection

**DOI:** 10.1007/s11748-013-0274-9

**Published:** 2014-12-01

**Authors:** Minoru Okamoto, Mutsuo Tanaka, Masanobu Ishii, Tsuyoshi Honda, Hidenobu Koga, Yuji Miyao, Kazuteru Fujimoto, Toshihiko Murayama

**Affiliations:** 1 Department of Cardiovascular Surgery, National Hospital Organization Kumamoto Medical Center, 1-5 Ninomaru, Chuou-ku, Kumamoto, 860-0008 Japan; 2 Department of Cardiovascular Medicine, National Hospital Organization Kumamoto Medical Center, Kumamoto, Japan; 3 Department of Pathology, National Hospital Organization Kumamoto Medical Center, Kumamoto, Japan

**Keywords:** Coronary artery dissection, Myocardial infarction, Coronary artery bypass grafting, Percutaneous coronary intervention

## Abstract

Spontaneous coronary artery dissection is rare and usually affects younger women in the peripartum period. Here, we report an interesting case of a 34-year-old woman with spontaneous coronary artery dissection that occurred 1 month after childbirth. Emergency coronary angiography showed stenosis of the left anterior descending artery, but immediately afterwards, a new occlusion of the right coronary artery occurred. Intravascular ultrasound was used to image both right and left coronary arteries. The new occlusion of the right coronary artery was probably iatrogenic, but the left coronary artery occlusion was spontaneous. The patient underwent percutaneous coronary intervention in the right coronary artery because of her unstable hemodynamic condition. Revascularization of the left coronary artery was performed by bypass grafting. The patient was discharged on postoperative day 30. As the optimal treatment for spontaneous coronary artery dissection has not yet been established, treatments should be based on the patient’s clinical presentation.

## Introduction

Spontaneous coronary artery dissection (SCAD) is a rare, but often fatal cause of acute coronary syndrome (ACS), with an incidence of 0.07–1.1 % [1, 2]. SCAD tends to occur in younger women and has been reported to be associated with a peripartum or postpartum status [1–3]. However, the optimal treatment of SCAD has not yet been established [1, 2]. Here, we report a case of SCAD that required percutaneous coronary intervention (PCI) and coronary artery bypass grafting (CABG), owing to a new occlusion and dissection detected by coronary angiography (CAG).

## Case

**Fig. 1:**
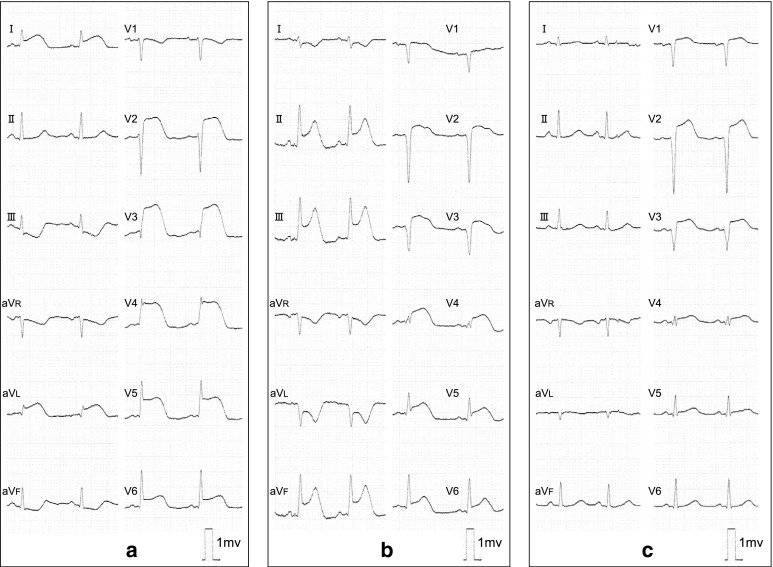
Electrocardiographic changes over time. **a** Initial ST elevation in leads I, aVL, and V2–4. **b** During the catheter procedure, new ST elevation in leads II, III, and aVF. **c** After the catheter procedure, improvement in ST elevation in leads I, aVL, and V2–4

**Fig. 2:**
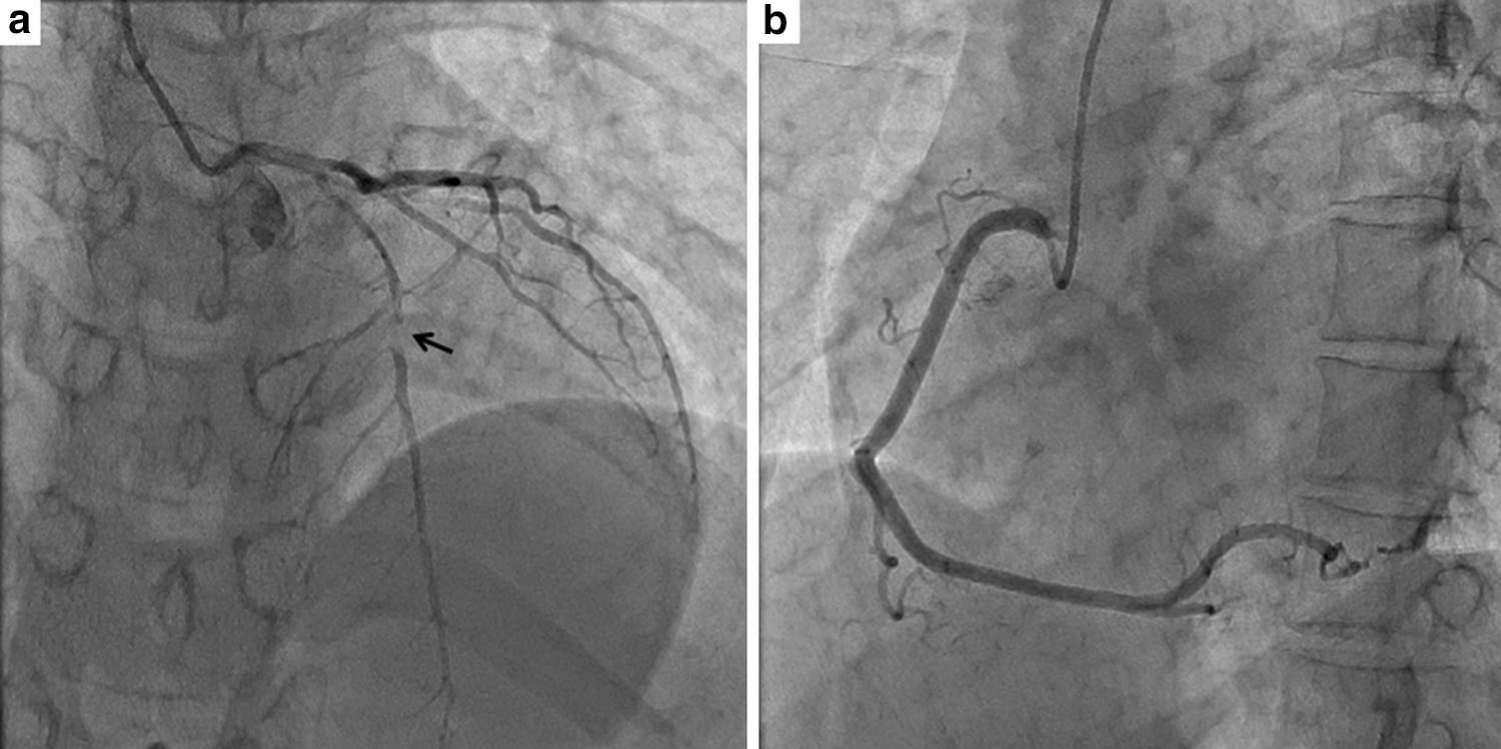
Initial angiogram. **a** Left coronary artery: the stenotic lesion can be seen in the left anterior descending artery (*arrow*). **b** Right coronary artery: no observable stenosis

**Fig. 3:**
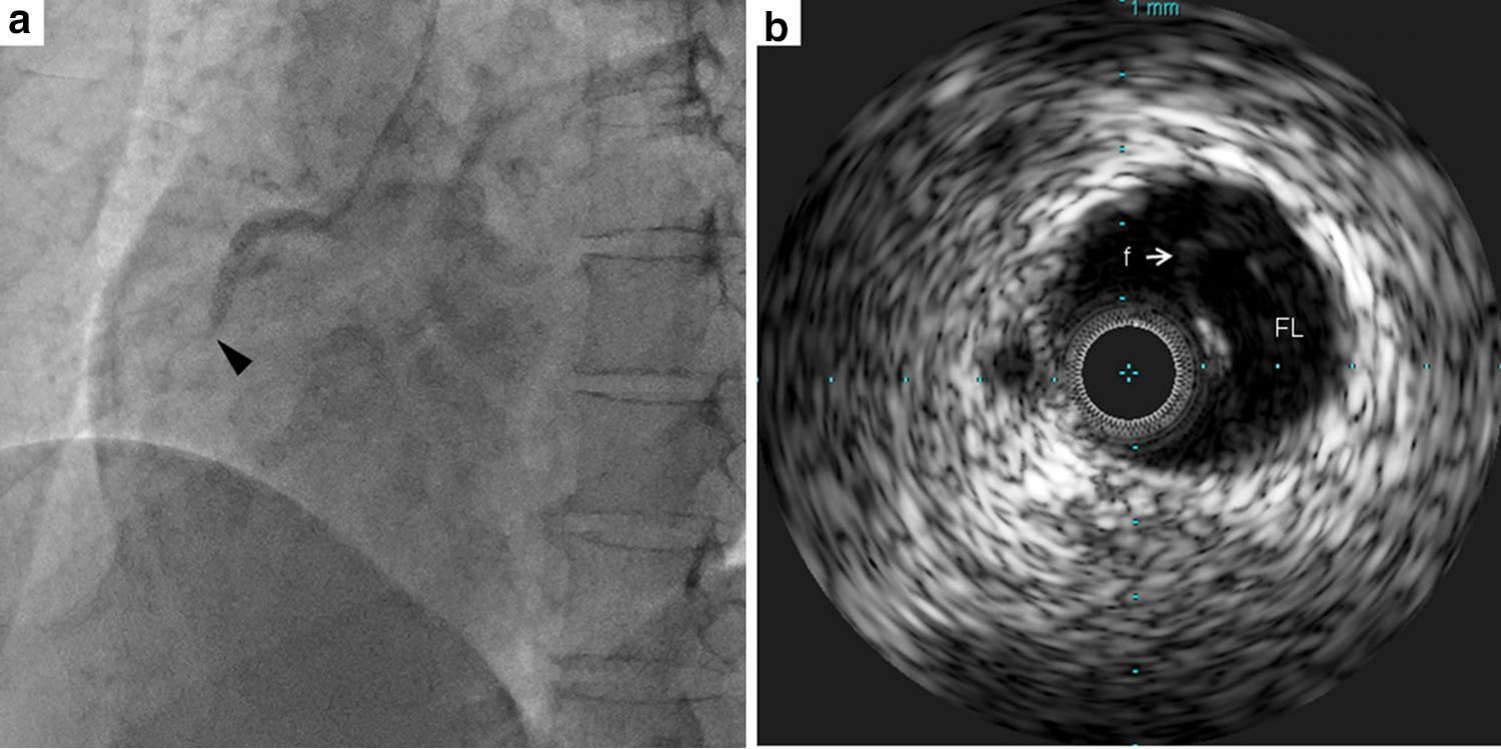
Angiogram and intravascular ultrasound of the right coronary artery after the electrocardiographic changes. **a** Angiogram: a new occlusion can be observed in the left anterior descending artery (*arrowhead*) after the intravascular ultrasound. **b** Intravascular ultrasound showing the false lumen and flap of the right coronary artery. *FL* false lumen, *f* flap

**Fig. 4:**
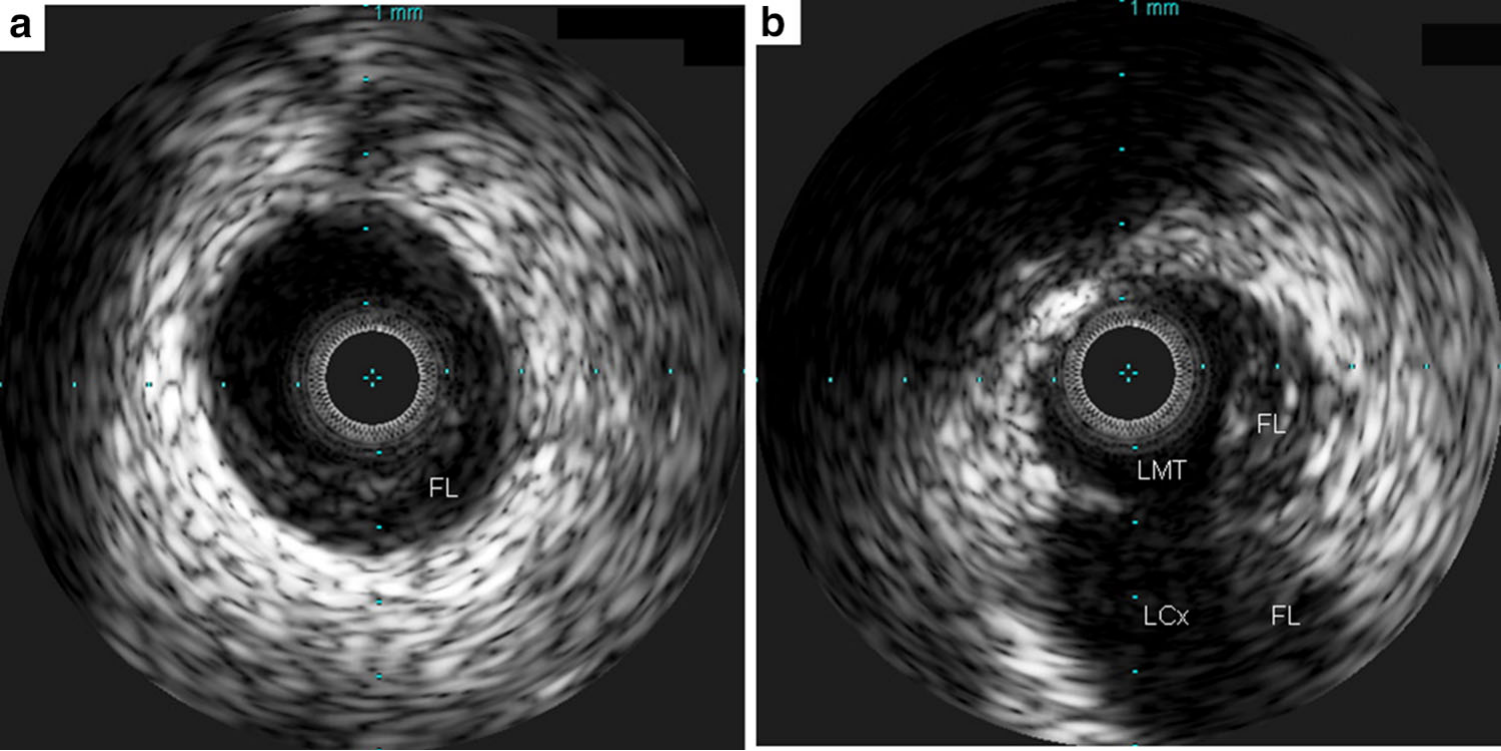
Intravascular ultrasound of the left coronary artery. **a** Left anterior descending artery showing the false lumen. **b** Left main trunk: the false lumen and the dissection extending to the left circumflex artery. *FL* false lumen, *LCx* left circumflex artery, *LMT* left main trunk

**Fig. 5:**
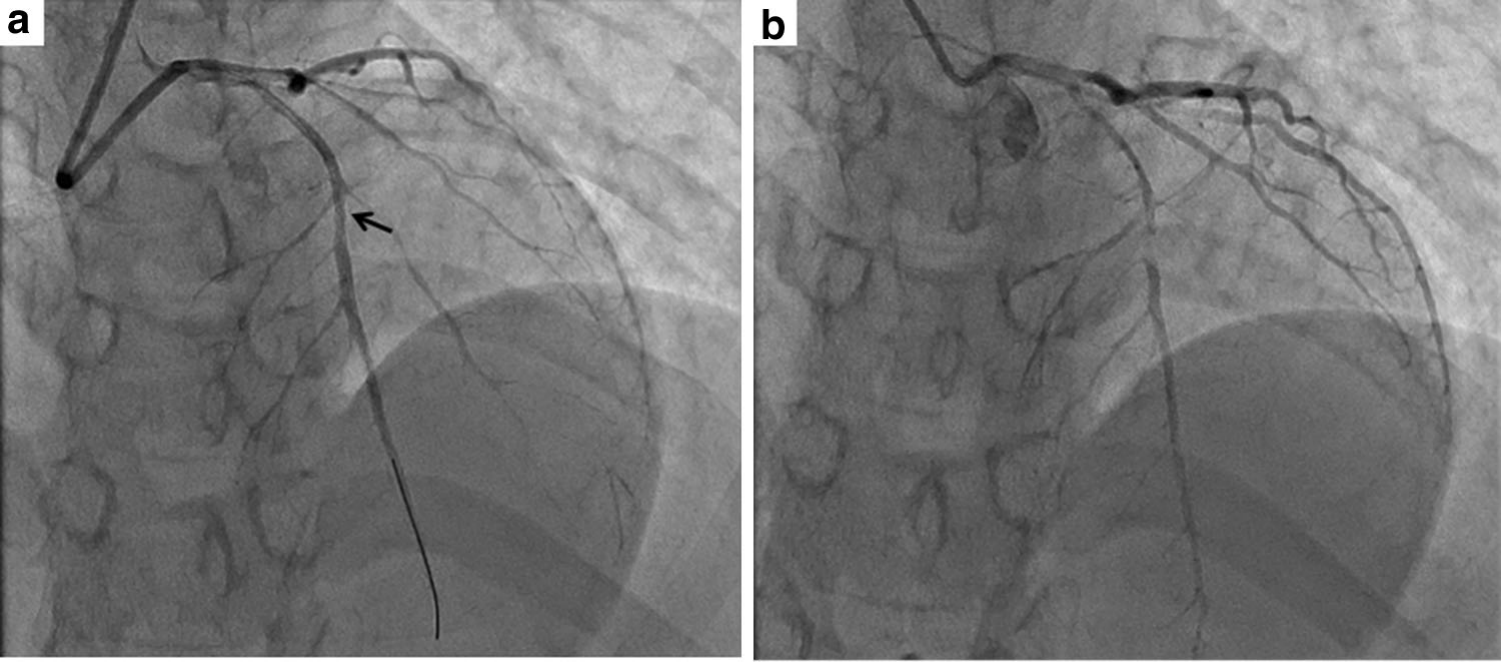
Angiogram of the left coronary artery before and after the intravascular ultrasound (IVUS) examination. **a** After IVUS, the stenosis in the left anterior descending artery improved slightly (*arrow*), despite the insertion of a guide-wire. **b** Before the IVUS, see legend of Fig. 2a

The patient was a 34-year-old woman who presented with chest pain to our hospital, 1 month after the birth of her second child. She had a history of gestational diabetes mellitus, but it was well controlled by diet alone. A 12-lead electrocardiogram (ECG) recorded on admission demonstrated ST elevation in leads I, aVL, and V2–4 (Fig. 1a). There was no physical evidence or family history of connective tissue disease, such as Marfan syndrome or Ehlers-Danlos syndrome (EDS). Initially, CAG of the left coronary artery indicated significant stenosis of the left anterior descending artery (LAD; Fig. 2a). Subsequently, CAG of the right coronary artery (RCA) was performed; it indicated that the artery was normal (Fig. 2b). Intravascular ultrasound (IVUS) and PCI were initiated. However, immediately afterwards, the ECG showed ST elevation in leads II, III, and aVF (Fig. 1b), and the patient’s systolic blood pressure dropped to 60 mmHg. An additional CAG demonstrated a new occlusion in the RCA (Fig. 3a), and the IVUS revealed RCA dissection (Fig. 3b). Since the RCA dissection occurred after the first CAG of the RCA, it was judged to be iatrogenic. Because of the patient’s unstable hemodynamic state, PCI was performed in the RCA using bare-metal stents. The PCI was successful, and her hemodynamic status improved. Subsequently, an IVUS examination of the LAD artery was performed and revealed the dissection extending to the left main trunk (LMT; Fig. 4). However, after the IVUS, the LAD artery stenosis improved slightly (Fig. 5). The thrombolysis in myocardial infarction (TIMI) flow score in the LAD artery improved to grade 3, the ST elevation diminished (Fig. 1c), and the patient’s hemodynamic state improved. Instead of PCI, CABG was planned for the LAD artery because of the residual stenosis in that artery. After the completion of CAG, levels of the cardiac enzymes, creatine kinase and creatine kinase-MB, were measured every 6 h. The enzyme levels first peaked at 6 h and then increased again 12 h later. The patient also complained of chest discomfort. Although the ECG changes were not significant compared to those after the PCI, emergency CABG was planned. An intra-aortic balloon pump was not used preoperatively because of possible hematoma migration to a false lumen and because connective tissue disease had not been completely excluded. With regard to the surgical intervention, off-pump CABG was planned taking account of the problem of possible connective tissue disease that might cause aortic dissection. Although there was a dissection in the LMT, revascularization of the left circumflex artery (LCx) was not planned, because no significant stenosis was observed from the LMT to the LCx, additionally TIMI flow and lateral wall motion of left ventricle were normal.

Surgery was performed via a median sternotomy. The pericardial effusion was serous, and the anterior wall of the left ventricle was dark red. However, the other ventricular walls were normal. Revascularization of the LAD artery using the in situ left internal thoracic artery (LITA) was planned, and off-pump CABG was performed. After the LITA was harvested, two stay sutures were placed on the posterior side of the pericardium to enable visualization of the LAD artery. Temporary occlusion sutures were placed between the proximal and distal sides of the anastomosis, and the LITA was anastomosed in an end- to- side manner. A skin biopsy was also performed at the incision site.

Pathological examination using the Elastica van Gieson stain revealed that the elastic fibers in the dermis were shortened and fragmented. However, the LITA specimen appeared normal. Thus, EDS type IV was excluded.

The patient’s postoperative course was uneventful, and she was discharged on postoperative day 30.

## Discussion

Coronary artery dissection can be primary or secondary: primary dissection occurs spontaneously, whereas the latter is a consequence of aortic dissection, an iatrogenic cause (such as PCI or cardiac surgery), or chest trauma [4]. Considering the types of SCAD mentioned above, the dissections in our patient were secondary (iatrogenic) in the RCA and spontaneous in the left coronary artery.

Approximately 70 % of SCAD patients are reported to be female, of whom approximately 30 % usually present in the peripartum period [5, 6]. Adam et al. [2] suggested that increased shear stress in vessels may play a role in SCAD because of the high cardiac output in pregnancy. Other risk factors for ACS are also associated with pregnancy, including hypertension, thrombophilia, and diabetes [7]. In our case, the patient had a history of gestational diabetes mellitus, which might have influenced the dissection. Among younger patients, connective tissue disease, including Marfan syndrome or EDS, should be considered. However, pathological studies of patients who died because of SCAD indicate that cystic median necrosis, often observed with connective disease, is rare. In the present case, the physical examination results and family history were negative for EDS type IV [8], as were the results of the pathological examination of the skin and LITA. Eosinophilic infiltration into the adventitia and injury of the vasa vasorum have also been observed in some cases of SCAD [3].

Although the culprit lesion was in the LAD artery in our case, the treatment plan was changed owing to the sudden RCA occlusion. The stenosis severity, LAD artery TIMI flow grade, and ECG changes improved after IVUS. Although the reason for this improvement was not clear and definitive evidence was not found, the IVUS procedure including guide-wire manipulation, might have been implicated in the deformation of the false lumen of the LAD, as IVUS was the only procedure that was performed before and after the improvement of LAD stenosis. In this regard, Adlam et al. [2] have illustrated the theory of propagation of a false lumen during stenting. Based on these considerations, we speculated that the IVUS catheter or the guide-wire compressed the false lumen, and this resulted in the improvement of the LAD stenosis in our case.

The sudden occlusion of the RCA was attributed to injury or exacerbation during the angiography procedure.

The optimal treatment for SCAD has not yet been established [1, 2]. Tweet et al. [1] reported that all patients treated with initial conservative therapy had a benign hospital course, whereas PCI was associated with elevated rates of technical failure. Misplacement of the guide-wire or displacement of the stent into the false lumen could reduce coronary flow [1, 2] and limit the effectiveness of PCI. In contrast, CABG as an initial strategy has been associated with a good short-term outcome, but the long-term occlusion rate of the bypass graft was reported to be high [1]. Because the pathophysiological mechanism of SCAD is complex, treatment should be selected according to the patient’s clinical presentation. Furthermore, given that the recurrence rate of SCAD is reported to be 17 %, and that it is observed exclusively in women [1], women treated for primary SCAD should be monitored carefully for recurrence during long-term follow-up.

## Conclusion

We have described a patient with SCAD who was treated with both PCI and CABG. SCAD is a rare, but life-threatening disease. Although PCI is less invasive than CABG, the high complication rate of PCI should be considered. A treatment plan, including conservative therapy, should be chosen after careful consideration, taking into account the patient’s condition and the results of CAG and IVUS examinations.
